# Supplementary material for: Deep learning enhanced Rydberg multifrequency microwave recognition
Source: Nat Commun. 2022 Apr 14;13:1997. doi: 10.1038/s41467-022-29686-7 (PMC9010414; doi:10.1038/s41467-022-29686-7)
Supplement: Supplementary file 1 — Supplementary Information [file 41467_2022_29686_MOESM1_ESM.pdf]

Supplementary materials for: Deep learning enhanced Rydberg multifrequency microwave recognition

Zong-Kai Liu[1,2], Li-Hua Zhang[1,2], Bang Liu[1,2], Zheng-Yuan Zhang[1,2], Guang-Can Guo[1,2], Dong-Sheng Ding[1,2,†], and Bao-Sen Shi[1,2,‡]

[1] *Key Laboratory of Quantum Information, University of Science and Technology of China, Hefei, Anhui 230026, China. and*
[2] *Synergetic Innovation Center of Quantum Information and Quantum Physics, University of Science and Technology of China, Hefei, Anhui 230026, China.*

(Dated: March 14, 2022)

† Corresponding Author: dds@ustc.edu.cn
‡ Corresponding Author: drshi@ustc.edu.cn

Supplementary Figures

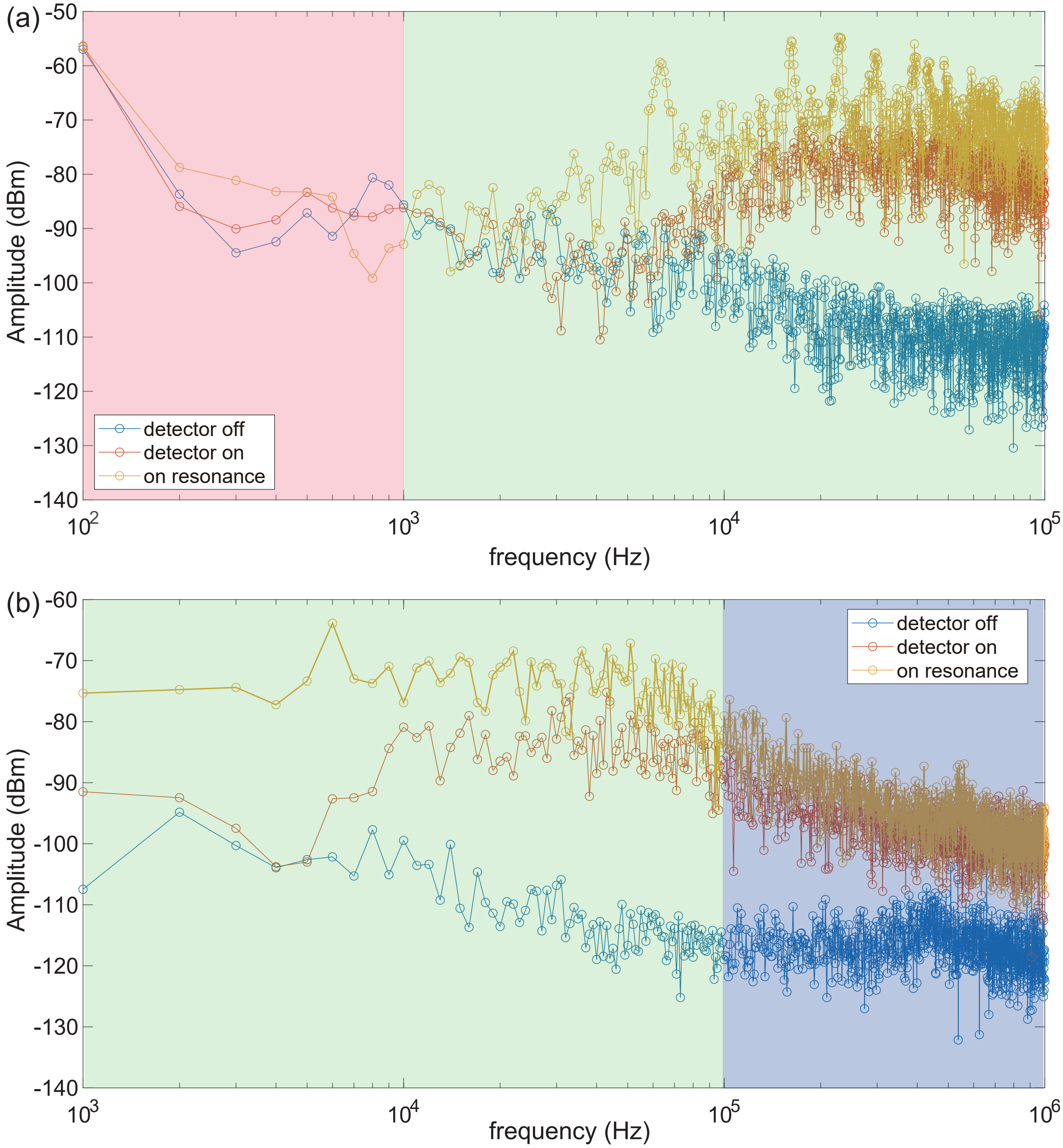

Supplementary Figure 1. **Systematic noise spectra.** Panels (a) and (b) illustrate systematic noise spectra within different range, i.e., (a) 0.1 kHz to 0.1 MHz and (b) 1 kHz to 1 GHz. The blue curve is the spectrum measured when the differential photodetector was switched off. The red curve is the spectrum measured when the detector was switched on but without a light signal. The yellow curve is the spectrum measured with the EIT configuration but without the microwave signal. The resolution bandwidth and the video bandwidth of the spectrum analyzer are both 10 Hz, and the attenuation is 10.

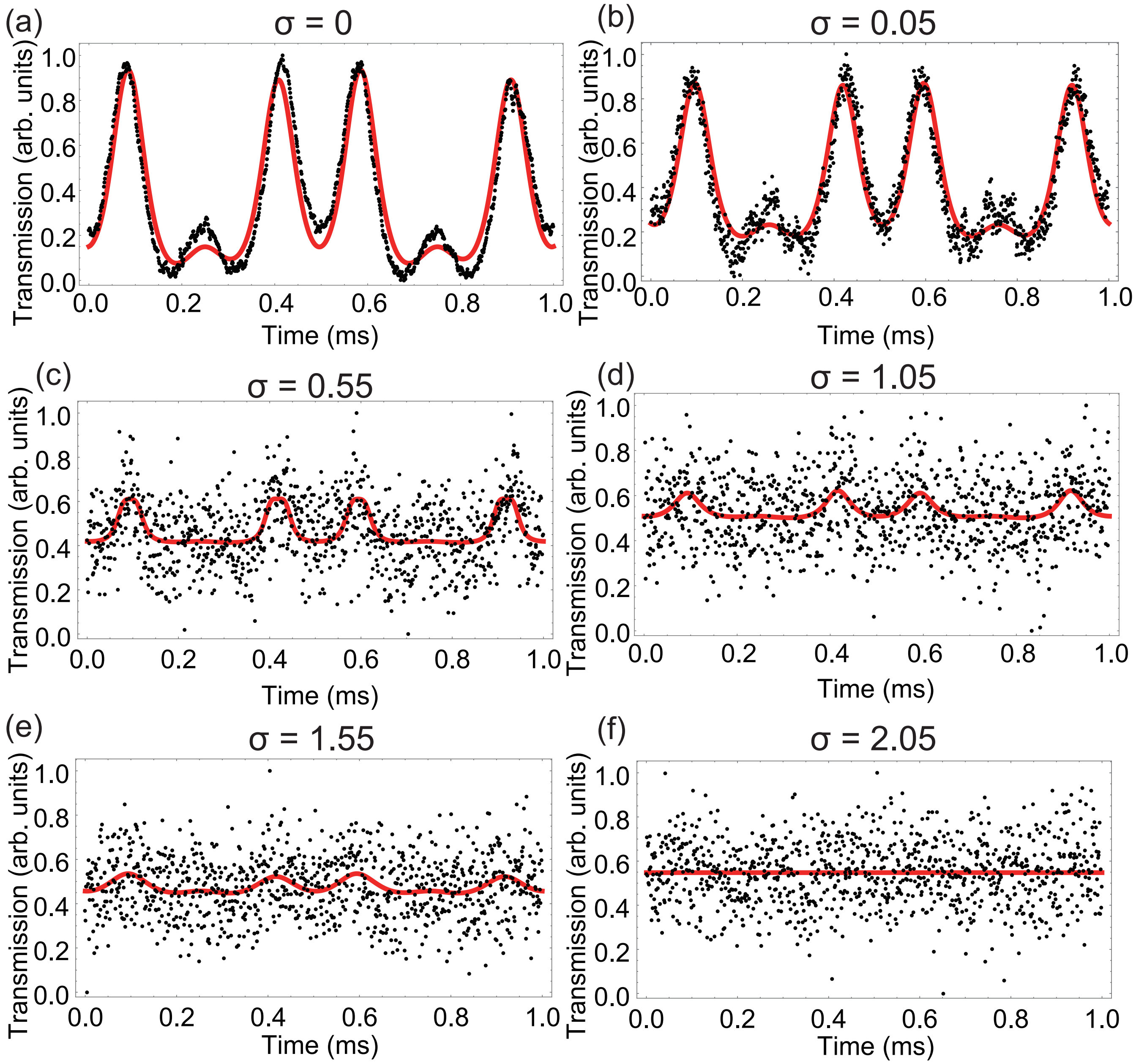

Supplementary Figure 2. **Fitting curve for the master equation on data with additional white noise.** The standard deviation values of the white noise are $\sigma = 0$ (a), 0.05 (b), 0.55 (c), 1.05 (d), 1.55 (e), and 2.05 (f), respectively. The prediction results are (0, 0, π, 0), (0, 0, π, 0), (0, 0, π, 0), (0, 0, π, 0), (0, 0, π, 0), and (0, 0, 0, 0), while the ground truths are both (0, 0, π, 0).

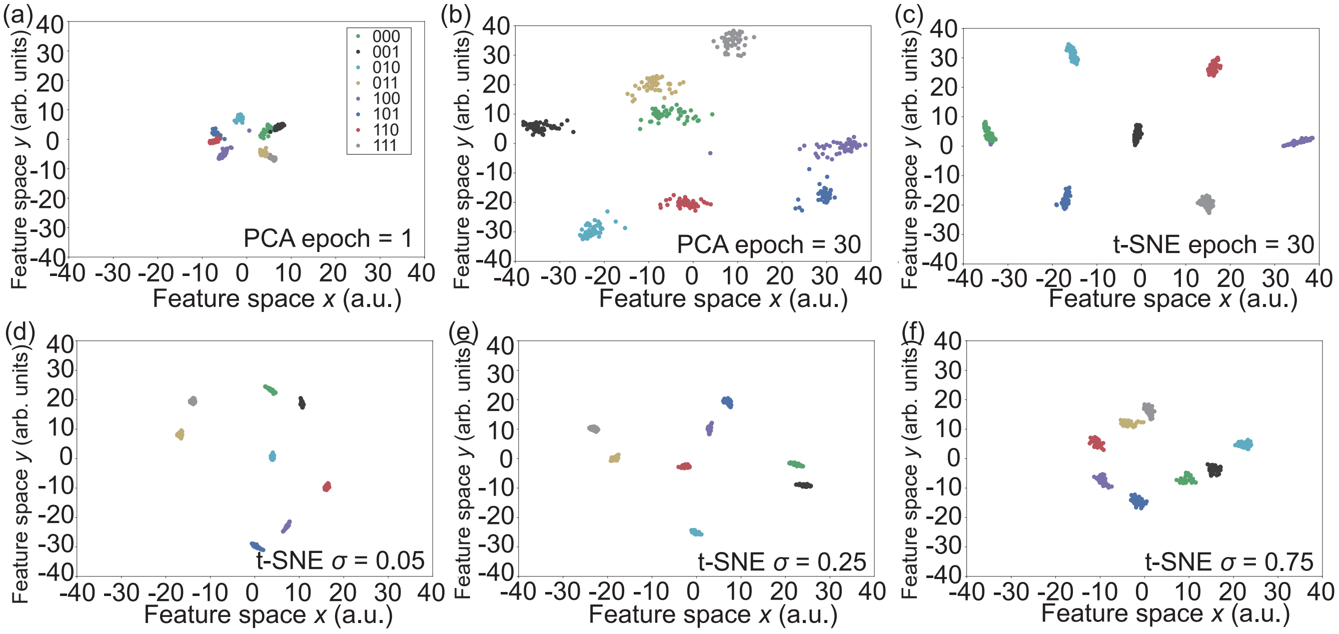

Supplementary Figure 3. **Visualization of the intermediate results of multiple inputs.** Dimensionality reduction methods are adopted. Each colored point represents a signal carrying a 3-bit message, and the same color data points carry the same message. This two-dimensional space is the feature space, where the points carrying the same message are clustered together and those carrying different messages are separated. After 1-epoch (a) and 30-epoch (b) training, the data are fed into the deep learning model and the outputs of the max-pooling layer are handled in principal component analysis. In (c) another method (T-distributed Stochastic Neighbor Embedding) is adopted to deal with the intermediate results of the 30-epoch model. (d–f) t-SNE results with the standard deviation of additional noise $\sigma = 0.05, 0.75$ and 0.95.

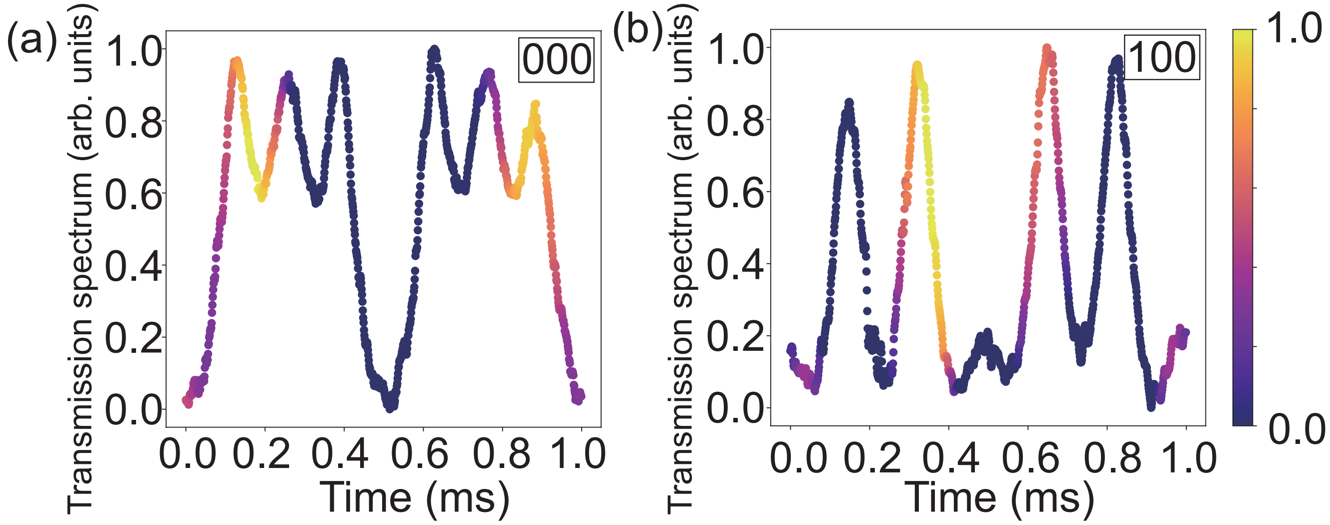

Supplementary Figure 4. **Heat map of the model for singular data.** The color represents how many focuses the model has on the local area of the data. The model recognizes the transmission spectrum via the area with bright color. (a) and (b) are heat maps for the data carrying messages “000” and “100”, respectively.

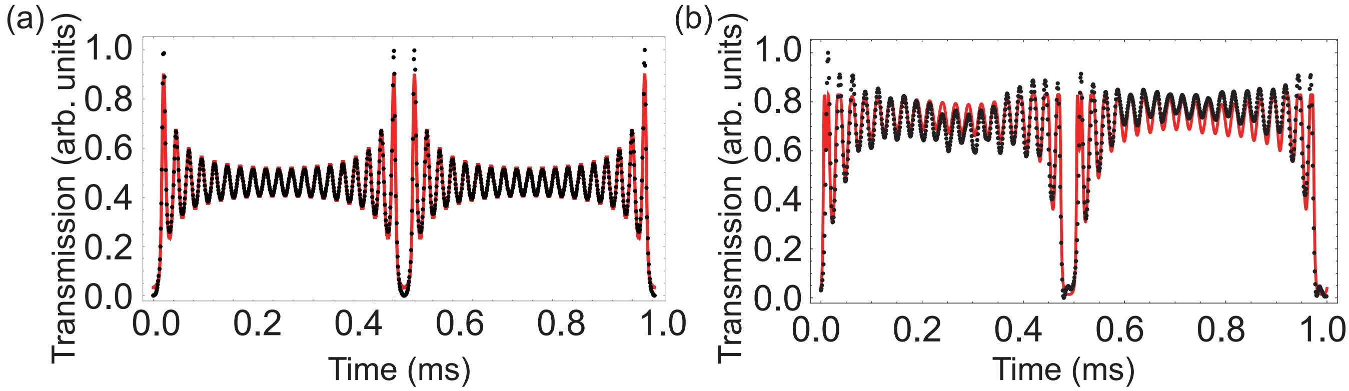

Supplementary Figure 5. **Using the master equation to fit the theoretical curve (a) and experimental curve (b).** The theoretical curve is generated by the master equation. In (a), the master equation fits the data well, whereas in (b), the master equation fits the data poorly.

Supplementary Notes

Supplemetary Note 1: Noise analysis

The systematic noise spectra are shown in Supplementary Fig. 1. The systematic noise comes from two sides: the first type is from the exterior, such as 1/f noise of electric circuits, background electric noise, and the noise from the mechanical vibration, etc.; the second type is from the interior (from atoms in the vapor cell) such as the transit noise due to thermal atoms. As stated in the literature [1], there are different types of noise for different frequencies. For frequency below 1 kHz, there is 1/f noise. Then, for a higher frequency range (1 kHz∼100 kHz), there is noise from atomic transitions out from and into the light area. At much higher frequencies (100 kHz∼1 MHz), the noise comes from the control systems of the lasers. During the experiments, we found that our deep learning model was robust with respect to these noise types. In fact, the deep learning model extracts the signal from noisy channel with high accuracy regardless of where the noise come from.

Under the systematic noise and the additional noise, the transmission spectrum are shown in Supplementary Fig. 2, where the additional noise is increased by adjusting its standard deviation σ. The fitting results of the master equation are also shown, which demonstrate that for larger noise the fitting curve of the master equation is distinct from the signal.

Supplemetary Note 2: Visualization the intermediate results of the deep learning model

After passing through the max-pooling layer, the data treated in the deep learning model are visualized directly, as shown in Fig. 7 of the main text. The data are expanded by the one-dimensional convolution kernel from one dimension to 20 dimensions. These new dimensions act as new features of the data. The feature space is then provided after the features of the intermediate results are reduced through principal component analysis (PCA) and T-distributed Stochastic Neighbor Embedding (t-SNE) [2]. PCA is a method of linearly reducing the dimensionality to project data to a lower dimensional space whereas t-SNE is a method of nonlinearly reducing the dimensionality. The feature space is obtained after the intermediate results are fed into these new models (even a linear method such as PCA). Signals carrying the same message are inclined to be together whereas those carrying different messages are separated, as shown in Supplementary Fig. 3. The features captured by the deep learning model are thus transferable and compatible with other models. Supplementary Fig. 3 also presents dimensionality reduction results for noisy data, showing that the capturing of features by the deep learning model is robust against additional noise.

We then refer to the Grad-CAM method, which is popular in the field of computer vision and used to highlight where the focus of a model is on an image [3]. Heat maps are presented in Supplementary Fig. 4, where the transmission spectrum and the model's focus are given. The area by which the deep learning model recognizes the signal from the heat maps is obvious. This visualization method obtains a perspective of how the deep learning model handles the signal.

In short, the model has learned the feature space and to focus on special areas after being trained, which is one of the reasons for why the deep learning model performs so well.

Supplemetary Note 3: The fitting curves with the master equation

Supplementary Fig. 5 shows the fitting of the theoretical and experimental curves with the master equation. If there is no internal or external noise, the master equation fits the spectrum perfectly, as shown in Supplementary Fig. 5(a). However, when the noise occurs in the experiment, the master equation performs poorly; see Supplementary Fig. 5(b). Meanwhile, in Fig. 2(e) of the main text, we presented the performance of the deep learning model being trained and tested on the data with biased additional noise. In the last row of Fig. 2(e) of the main text, the model is trained on a training set without additional noise and is tested on a test set with the additional noise that it has never seen before. However, the deep learning model performs well. Once the deep learning model has learned the signal pattern from the training set without additional noise, it extracts the features from the noisy test set even with the noise it has not seen before. Indeed, to further increase the accuracy of the master equation, prior knowledge must be involved to limit the initial values. In contrast, the deep learning model learns to decode the signal by itself without human intervention.

Supplementary References

[1] M. Jing, Y. Hu, J. Ma, H. Zhang, L. Zhang, L. Xiao, and S. Jia, Atomic superheterodyne receiver based on microwave-dressed Rydberg spectroscopy, Nature Physics **16**, 911 (2020).

[2] F. Pedregosa, G. Varoquaux, A. Gramfort, V. Michel, B. Thirion, O. Grisel, M. Blondel, P. Prettenhofer, R. Weiss, V. Dubourg, J. Vanderplas, A. Passos, D. Cournapeau, M. Brucher, M. Perrot, and E. Duchesnay, Scikit-learn: Machine learning in Python, Journal of Machine Learning Research **12**, 2825 (2011).

[3] R. R. Selvaraju, M. Cogswell, A. Das, R. Vedantam, D. Parikh, and D. Batra, Grad-cam: Visual explanations from deep networks via gradient-based localization, International Journal of Computer Vision **128**, 336–359 (2019).
